# Exploring the role of interpersonal contexts in peer relationships among autistic and non-autistic youth in integrated education

**DOI:** 10.3389/fpsyg.2022.946651

**Published:** 2022-07-22

**Authors:** Yu-Lun Chen, Maxwell Schneider, Kristie Patten

**Affiliations:** ^1^Department of Occupational Therapy, New York University, New York, NY, United States; ^2^Fresh Path NY, New York, NY, United States

**Keywords:** double empathy problem, integrated education, inclusion, peer interactions, relationships, social behaviors, network analysis, homophily

## Abstract

The double empathy problem theory posits that autistic social difficulties emerge from an interpersonal misalignment in social experiences and expectations between autistic and non-autistic people. Supporting this, emerging research reveals better social outcomes in interactions within than across neurotypes among autistic and non-autistic people, emphasizing the need to examine the role of the interpersonal context in autistic social outcomes. However, research on peer relationships among autistic youth primarily focuses on individual characteristics in isolation from the interpersonal context. To address this, this preliminary study explored the effects of student-peer neurotype match on peer relationships among autistic and non-autistic youth in an integrated educational setting. We plotted the peer relationship networks among youth in a school club based on systematic observations of peer interactions over eight 45-min sessions. Descriptive network statistics (node degree and strength) showed that both autistic and non-autistic youth had more and stronger peer relationships with their same- than cross-neurotype peers. Assortativity coefficients revealed a tendency for youth to connect with peers of the same neurotype, rather than with peers with similar social popularity or activity. We further modeled the effects of student-peer neurotype match on peer relationships using exponential random graph models. The findings suggested that student-peer neurotype match predicted the total strength of peer relationships above and beyond the effects of student neurotype, individual heterogeneity in social popularity and activity, and the tendency of mutuality in social relationships. We discussed the strengths and limitations of this study and the implications for future research and inclusion practice.

## Introduction

Autistic youth^1^ learning in integrated educational settings experience significant challenges in peer relationships (Williams et al., 2019). Compared to their non-autistic peers, these youth engage in fewer peer interactions and friendships (Humphrey and Symes, 2011; Kasari et al., 2011; Locke et al., 2016) and experience more peer rejection and victimization (Cook et al., 2016; Maiano et al., 2016; Cresswell et al., 2019). Autistic students in integrated classrooms have fewer peer connections than non-autistic students and tend to be peripheral, or even isolated in their classroom social networks (Chamberlain et al., 2007; Rotheram-Fuller et al., 2010; Kasari et al., 2011; Locke et al., 2012). However, social connections and friendships are crucial to the well-being and mental health of autistic people (Mazurek, 2014; Hedley et al., 2018), and thus there is a crucial need to support peer relationships in autistic youth.

Research on autistic peer relationships primarily focuses on individual factors, reflecting an assumption that individual social deficits lead to the social challenges experienced by autistic people. However, peer relationships are interpersonal processes between an autistic student and their peer, where both people collectively shape the outcomes. Recognizing this, recent theories emphasize the need to situate autistic social interactions in the interpersonal context (Milton, 2012; De Jaegher, 2013; Bolis et al., 2017; Milton et al., 2020). For example, the Double Empathy Problem Theory reconceptualizes autistic social challenges as interpersonal barriers and disjuncture in reciprocity between people with different social norms and expectations, such as between autistic and non-autistic people (Milton, 2012; Milton et al., 2020). With different social disposition and perceptions, social interaction between autistic and non-autistic people are susceptible to misunderstandings or gaps in mutual understanding, which is a “double empathy problem” as both people experience it instead of a singular problem in autistic people.

Supporting the double empathy theory, studies have shown that matched-neurotype social interaction within both autistic and non-autistic dyads experience more positive interpersonal rapport (Crompton et al., 2020a), greater interest in future interaction (Morrison et al., 2020b), and more accurate information transfer (Crompton et al., 2020b) than cross-neurotype pairs. Autistic individuals have also reported feeling more comfortable, understood, and accepted in interaction with their autistic than non-autistic family and friends and have connected their social experience with non-autistic people with pressure to conform to non-autistic social norms (Crompton et al., 2020c).

Collectively, these studies highlight the need to examine autistic social connections through a bidirectional methodology that further considers the influences of peer factors, such as peer neurotype. However, there remains a limited understanding of the effects of peer neurotype on autistic peer relationships. Emerging evidence shows that autistic youth in integrated education are more likely to interact with their same-neurotype (i.e., autistic) peers than cross-neurotype ones, and this preference for same-neurotype peers is also found in non-autistic youth (Chen et al., 2021). It is, however, unknown whether the effects of peer neurotype extend beyond peer interactions and transfer into peer relationships among autistic and non-autistic youth. Exploring the role of student-peer neurotype match in peer relationships would improve our understanding of how autistic and non-autistic youth navigate and develop connections, providing critical information regarding the social inclusion challenges experienced by autistic youth and informing supportive interventions to address these challenges.

This observational study aimed to examine the effects of student-peer neurotype match on peer relationships among autistic and non-autistic youth in an inclusive educational setting, using social network analysis. We observed social interactions among autistic and non-autistic youth in an inclusive school club (Maker Club) where peer interactions were not intervened. To measure observable peer relationships, we operationalized peer relationships as connections (or social ties) between a pair of youth as indicated by frequent social interactions observed between the dyad across multiple sessions. A student’s peer relationships were measured in two aspects: the quantity (the number of peers they interact with) and the strength of their connections (how frequent were the interactions) with others.

To investigate the role of student-peer neurotype match in peer relationships, we addressed three research questions: (1) Does student-peer neurotype match predict the quantity and strength of peer relationships in autistic and non-autistic youth? (2) Do autistic and non-autistic youth tend to develop relationships with their same-neurotype peers? Alternatively, do they tend to connect with peers who share similar social status (i.e., social popularity and activity), which is common in interpersonal social networks (Newman, 2002, 2003)? (3) Does student-peer neurotype match predict the strength of peer relationships above and beyond the effects of student neurotype, individual heterogeneity in social popularity and activity, and the tendency of mutuality in social relationships? Mutuality refers to the tendency of reciprocating social connections (i.e., when A interacts with B, there is a higher chance for B to reciprocate this interaction), which is theoretically and intuitively expected in interpersonal social networks (Holland and Leinhardt, 1981). We included this effect in the network model because reciprocation in social interactions is a social norm that is commonly observed in social relationships.

Through these questions, we examined the Double Empathy Problem Theory, based on which we hypothesized the following. Research question (1): both autistic and non-autistic students would present more and stronger connections with their same-neurotype than cross-neurotype peers. This is because the theory posits that cross-neurotype dyads would experience more barriers to mutual understanding due to interpersonal differences in social perceptions and expectations. Research question (2): students would present a stronger tendency to connect with peers with the same neurotype rather than with those with similar social status. Research question (3): Student-peer neurotype match would significantly predict the strength of peer connection above and beyond the effects of student neurotype, social mutuality, and individual differences in social activity and popularity.

## Methods

### Research design and context

Through systematic observations of peer interaction behaviors in an inclusive school club (the Maker Club), we plotted the peer relationship networks among autistic and non-autistic youth over five months. The Maker Club was an inclusive extracurricular program designed to facilitate STEM learning in autistic and non-autistic youth at a public middle school in a large, urban area of the northeastern United States. At the beginning of the school year, all students in Grades 6-8 (12-14 years of age) were invited to enroll in the club, and twelve students volunteered to participate in the program.

The public school where the study took place was a part of the city’s specialized education program for autistic students. The program served autistic and non-autistic students in a reduced class-size Integrated Co-Teaching model with one special education teacher and one general education teacher. Staff received training in specialized teaching strategies for students on the spectrum. The curriculum followed the Common Core Learning Standards as in all schools of the New York City Department of Education. In addition to the academic curriculum, autistic students received social development intervention taught in small groups to support their social and emotional skills.

### Participants

Participants were the 12 students enrolled in the Maker Club, including six autistic and six non-autistic youth. Autistic students were all enrolled in the public school’s autism-inclusion program, which required them to: (1) have a diagnosis of Autism confirmed by an up-to-date evaluation of the Autism Diagnostic Observation Schedule by the Department of Education; (2) verbal language on or close to age level; (3) average to above-average intellectual functioning; and (3) academic skills on or above grade level. Table 1 presents participant demographics. All participants provided assent to participate in the study, and their parents provided written consent forms. The Institutional Review Boards in the university and the school district approved the study.

**TABLE 1:** Participant characteristics.

	Autistic (*n* = 6)	Non-Autistic (*n* = 6)
**Gender**		
Male	5	3
Female	1	3
Grade		
6th	3	5
7th	3	1
8th	0	0
Race/Ethnicity*		
American Indian or Alaska Native	0	1
Asian	1	0
Black or African American	2	3
Hispanic or Latinx	1	4
Pacific Islander or Native Hawaiian	0	1
White or Caucasian	2	1
Other	2	3

*Participants were allowed to select more than one ethnicity.

### Social behavior observations

We conducted social behavior observations based on video recordings to enable review of the data when needed, such as when interrater disagreement occurred. We video-recorded a total of 14 45-minute observation sessions over the research period. The recordings of six out of the 14 sessions were excluded because more than four students were absent, such as due to other school activities. Therefore, the final analysis included eight sessions where at least eight of the 12 students were present and observed.

We observed peer interaction behaviors in video recordings, coding each student’s social behaviors and the peers with whom they interacted. To facilitate accurate observations, all verbal behaviors (e.g., conversations) were transcribed verbatim, and non-verbal behaviors (e.g., gesturing) were described in transcriptions. We did not observe facial expressions and eye contact because students’ faces were blurred in the videos for deidentification. We also did not consider these behaviors reliable indicators for peer relationships, since eye contact and facial expressions do not necessarily reflect social motivation among autistic people (Jaswal and Akhtar, 2018). The observed social behaviors were classified as social initiation or responses. Social initiations were defined as a student’s attempts to begin a social sequence (e.g., starting a conversation or greeting a peer). Social responses were defined as social reactions to peers’ initiation or ongoing conversations (e.g., answering a question, granting a request, or joining a conversation). The sample interaction below illustrates the coding system (excerpted from Chen et al., 2021):

Ethan: You’re drawing Kirby? [coded as *social initiation*]

Mathew: (Nods) [coded as *social response*]

Ethan: And his eyes? [coded as *social response* as it is continuing the prior social exchange]

Mathew: Mhmm. That’s why I chose blue. [coded as *social response*.]

### Social network plotting

**FIGURE 1:**
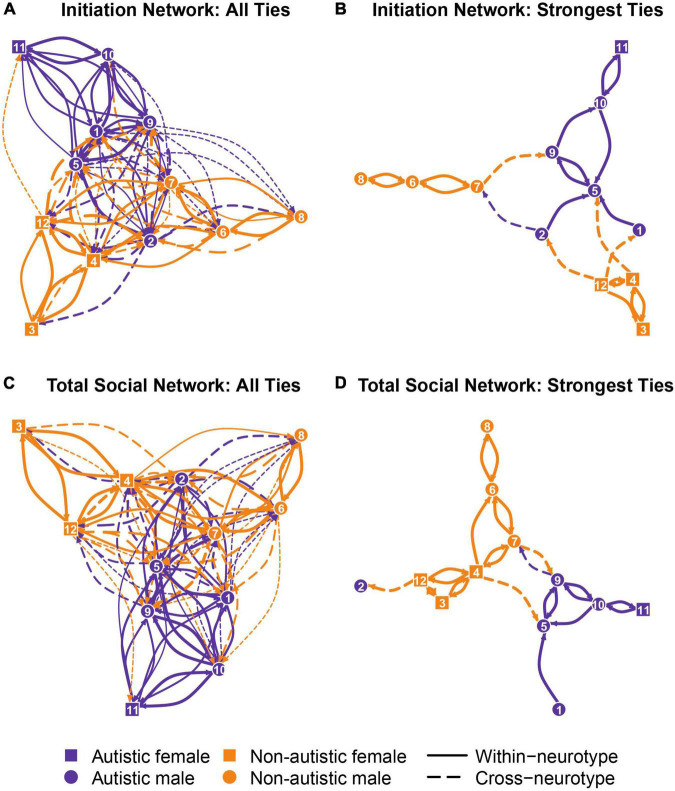
Mean social networks across observations. Each node represents a student in the club, and each edge between a pair of nodes represents the peer relationship between the dyad. Arrows represent the direction of the edges, pointing from the sender to the receiver of social interactions. The width of an edge indicates the strength of the social tie (the thicker the edge, the stronger the tie). **(A)** The network of social initiations among youth. **(B)** A subnetwork of panel A with only the strongest social ties. **(C)** The network of all social interactions (initiations and responses) among youth. **(D)** A subnetwork of panel C with only the strongest ties.

Based on the observed social behaviors, we plotted peer social networks in the eight club sessions. To illustrate this, Figure 1 presents the average social network across all sessions. In Figure 1, each node represents a student, while an edge between any two nodes represents the social tie (or peer relationship) between the two students. The absence of an edge between any pair of nodes suggests that no interactions occurred between the pair. Edges were directional, with the arrow of an edge pointing from the sender of social behaviors toward the receiver. Therefore, based on the directions of edges between two nodes, one can tell whether the peer relationship between the pair reciprocated.

Edges were weighted to indicate the strength of relationships, with thicker edges showing stronger relationships. To determine the strengths of peer relationships, we calculated social behavior rates (behavior count/observation length) observed between each dyad. Specifically, we categorized peer relationships in four levels based on the quartiles of social behavior rates across all students and sessions (1 = very weak relationship, as indicated by a social behavior rate ranked below the 25th percentile of all observations; 2 = weak relationship, between the 25th and 50th percentiles; 3 = strong relationship, between the 50th and 75th percentiles; and, 4 = very strong relationship, above the 75th percentile). For peer dyads with no observed social behaviors, the strength was assigned as zero (no edge).

In addition to the *total social networks* that included all social behaviors among the students, we further plotted the *social initiation networks* among the participants, which included only social initiation behaviors. These networks were generated to understand students’ preferences in the peers they chose to initiate interactions with. See Figure 1 for the average total social network (C) and initiation network (A) across observations.

### Descriptive network analysis

#### Node degree and node strength by student-peer neurotype

We quantified the quantity and strength of peer connections using two measures, node degree (Freeman, 1978) and node strength (Barrat et al., 2004). Node degree calculates the total quantity of connections each student had in a network, while node strength sums up the edge weights of the social ties connected to the node (i.e., the total strength level of a student’s all social connections). We calculated node degree and node strength for within- and cross-neurotype peer connections, with which we explored whether these measures differed by student and peer neurotypes using two mixed-effects negative binomial regression models. Node degree and node strength were the dependent variables, and independent variables included student neurotype (autistic vs non-autistic), student-peer match (within- vs cross-neurotype), student gender, and student-level random intercepts to control for the interdependency of repeated observations.

#### Network assortativity

We used assortativity coefficients (Newman, 2002, 2003) to quantify the tendency for youth to connect with peers who shared the same neurotype or similar social status. Specifically, we calculated assortativity coefficients based on three student attributes: (1) student neurotype, (2) student social popularity and activity as indicated by the number of peer connections (node degree), and (3) student social popularity and activity as indicated by the strength of peer connections (node strength). Assortativity coefficients range from –1 to 1, with positive values indicating a tendency for nodes of similar properties to connect and negative values suggesting a trend for nodes to connect with those with different properties.

### Social network modeling

#### Exponential random graph models

We used Exponential Random Graph Models (ERGMs) to investigate the effects of student neurotype and student-peer neurotype match on predicting peer connections besides the effects of social popularity and activity. ERGMs are statistical models for network structures that permit inferences about the formation of network ties by modeling the probability that a relation exists in an observed network as a generalized linear function of predictors (Robins et al., 2007; Goodreau et al., 2009; Lusher et al., 2013). Differing from generalized linear regression, ERGMs assume interdependency between social ties and consider individuals as actors in social relations instead of independent units of analysis (Goodreau et al., 2009; Lusher et al., 2013). This assumption reflects the theoretical view that social connections are interdependent and relational, making ERGMs a suitable tool to investigate social connections among autistic and non-autistic youth. Another strength of ERGMs is the capability of simultaneous modeling of actor covariates (e.g., student attributes), dyadic covariates (e.g., relationship attributes), and network structural effects (e.g., reciprocation of social ties), which allows flexible and powerful inferences.

We fitted ERGMs to predict peer connections with the following terms: student attributes including (1) student neurotype (autistic vs non-autistic) and (2) student gender (female vs male; students were asked whether they identified as male, female, or neither, and no student selected neither); a dyadic covariate of (3) student and peer neurotype match; as well as network structural effects including (4) an overall social connection effect, which resembles the intercept term in linear models, (5) mutuality (i.e., a tendency of reciprocal connection between students), and (6) social activity and popularity^2^. All terms were defined as valued ERGM terms, where the overall social connection effect was specified as the sum of levels of strength in all peer connections and the mutuality term was generalized by evaluating the minimum of the tie values in both directions between a pair (Krivitsky, 2012). Individual heterogeneity in social activity and popularity were specified as within-actor uncentered covariances of square roots of out- and in-dyad values, respectively, which increase with great heterogeneity (Krivitsky, 2012). Since levels of social connection strength were based on quartiles of social behavior rates, we defined the reference distribution of the network values as a discrete uniform distribution with an upper bound of four. As peer connections were sparse for some students, we added a zero-modification term to the model to capture the probability of no connection in a dyad. The identical model configuration was used on all four peer networks (i.e., initiation, response, high reciprocity, and low reciprocity networks) and across the eight observation sessions.

#### Meta-analysis across observations

Considering the social networks of each observation session as a snapshot of the club social network, we sought to examine the overall network structure and effects by summarizing the model estimates across observations and exploring between-observation variability. We conducted a meta-analysis over models across observations using a two-step weighted least squares regression, which is an established approach to combine micro-level ERGMs in a macro-level analysis (Lubbers, 2003; Snijders and Baerveldt, 2003) and resembles a meta-analysis on data from multiple experiments (Cochran, 1954; Hedges and Olkin, 2014). Based on the coefficient estimates and standard errors identified in models of all observations, we calculated the weighted least squares estimator for average parameter estimates and tested whether the estimated average predictor effects were zero using the *t*-ratio, defined as the ratio between average parameter estimate and the associated standard error (Lubbers, 2003; Snijders and Baerveldt, 2003). We excluded ERGMs in each observation that failed to converge or presented extremely high standard errors (≥ 5), as such estimates tended to be unreasonable and would affect meta-analysis (Lubbers, 2003; Snijders and Baerveldt, 2003).

We conducted all data analysis in R (R Core Team, 2020), using the package igraph (Csardi and Nepusz, 2006) for network visualization and descriptive analysis, lme4 (Bates et al., 2015) for mixed-effects modeling, ggplot2 (Wickham, 2016) for charting descriptive data, and ergm and statnet (Hunter et al., 2008; Handcock et al., 2020) for social network modeling.

### Community participation

The second author of the study was autistic and reviewed the research questions and methods of the study to ensure they reflected the interests of the autistic community. The autistic researcher contributed to the interpretation of the findings with their earlier experiences of peer engagement in inclusive education as well as their lived experience socializing with autistic and non-autistic people.

## Results

### Average peer connection networks

Figure 1 presents the average peer connection networks, including the initiation network (A), total social network (C), and simplified subnetworks of these two networks with only the strongest peer connections (B & D). All graphs consistently showed more within-neurotype than cross-neurotype connections in both autistic and non-autistic youth. The networks and subnetworks also presented assortative mixing by gender, where males and females formed different clusters, except for the only autistic female in the club, who had minimal interactions with other females (non-autistic). This autistic female was located at the periphery of all club networks because of her fewer and weaker peer connections.

### Node degree and node strength

*RQ 1) Does student-peer neurotype match predict the quantity and strength of peer relationships in autistic and non-autistic youth?*

Table 2 presents the mean node degree and node strength across eight observation sessions by student and peer neurotypes. Both groups showed higher node degree and stronger node strength in within-neurotype than cross-neurotype connections. Mixed-effects models showed that student-peer neurotype match was significantly associated with more social ties and stronger connection strengths when controlling for student neurotype and gender (Table 3), suggesting that students had more and stronger within-group peer connections than cross-group connections. Autistic neurotype did not significantly predict either the quantity or strength of connections in both networks.

**TABLE 2:** Node degree and node strength by neurotype match.

Neurotype Match	Autistic				Non-Autistic			
**Network**	**Within-Group**		**Cross-Group**		**Within-Group**		**Cross-Group**	
**Initiation**	***M***	***SD***	***M***	***SD***	***M***	***SD***	***M***	***SD***
Node degree	3.06	1.72	1.89	2.00	2.65	1.06	1.84	1.50
Node Strength	7.44	4.47	4.28	4.76	7.84	3.65	4.16	3.60
**Total**								
Node degree	4.39	2.00	2.53	2.30	3.14	1.29	2.46	1.79
Node Strength	11.11	6.26	5.72	6.08	9.51	3.75	5.57	4.45

Node degree and Node Strength were based on all social ties (received and sent ties).

**TABLE 3:** Effects of neurotype match on node degree and node strength in mixed-effects models.

Variable	(Intercept)		Autistic		Neurotype Match		Female	
**Network Measure**	**Estimate**	***SE***	**Estimate**	***SE***	**Estimate**	***SE***	**Estimate**	***SE***
**Initiation**								
Degree	0.64 ***	(0.12)	0.05	(0.12)	0.43 ***	(0.11)	−0.16	(0.14)
Strength	1.45 ***	(0.15)	−0.02	(0.15)	0.59 ***	(0.14)	−0.02	(0.17)
**Total**								
Degree	0.90 ***	(0.11)	0.14	(0.10)	0.41 ***	(0.10)	−0.22	(0.12)
Strength	1.72 ***	(0.14)	0.07	(0.14)	0.60 ***	(0.13)	−0.08	(0.16)

Significance levels: *** < 0.001, ** < 0.01, * < 0.05. All models were based on 146 observations in 12 student groups. Estimates reflect expected differences in log count of the number of social ties.

### Network assortativity

*RQ 2) Do autistic and non-autistic youth tend to develop relationships with their same-neurotype peers? Alternatively, do they tend to connect with peers who share similar social status?*

Table 4 presents the mean assortativity coefficients across observations, based on student neurotype, node degree, and node strength. In all networks and subnetworks, assortativity coefficients of student neurotype were greater than those of node degree and strength, suggesting that students had a stronger tendency to connect with those with their same-neurotype peers than peers with similar social status. Subnetworks of the strongest ties showed a greater tendency of assortative mixing by neurotype than the full networks.

**TABLE 4:** Assortativity coefficients by neurotype, node degree, and node strength.

	Neurotype		Node degree		Node Strength	
**Network**	**M**	**SD**	**M**	**SD**	**M**	**SD**
*Initiation (all ties)*	0.27	0.29	0.03	0.29	0.13	0.32
Strongest ties only	0.47	0.53	−0.14	0.44	−	−
*Total (all ties)*	0.24	0.25	0.06	0.13	0.07	0.21
Strongest ties only	0.49	0.54	0.15	0.62	−	−

A positive coefficient suggests a tendency for students with similar attributes to connect, while a negative coefficient suggests a tendency to connect with dissimilar peers. The value of a coefficient suggests the intensity of assortative/disassortative mixing, where a coefficient close to -1 or 1 suggests the greatest level of disassortativity or assortativity, respectively, while a coefficient close to 0 suggests no tendency of assortative mixing. For subnetworks of students’ strongest social ties, assortativity coefficients based on node strengths were not calculated because all ties were equally strong.

### Exponential random graph model estimates

*RQ 3) Does student-peer neurotype match predict the strength of peer relationships above and beyond the effects of student neurotype, individual heterogeneity in social popularity and activity, and the tendency of mutuality in social relationships?*

Table 5 presents the results of the meta-analysis of ERGMs across observations. Student neurotype was not significantly associated with peer connections in both the initiation and total networks. Student-peer neurotype match was significantly associated with a stronger total strength of peer connections in the total social network (average estimate = 0.16, odds ratio = 1.17, *p* < 0.001), suggesting that the odds for a within-neurotype peer dyad to form an extra connection or to increase the strength of an existing connection in one unit were 17% greater than that in a cross-neurotype dyad. The initiation network showed a similar trend that did not reach significance (average estimate = 0.17, odds ratio = 1.19, *p* = 0.069). The effects of neurotype match were above and beyond the effects of mutuality, gender, popularity, and activity.

**TABLE 5:** Effects of neurotype match on peer connection in ERGMs.

Term	Average Estimate	SE	*t*-ratio	*p* (*t*)
***Initiation*** (*N* = *7)*				
Sum (Intercept)	−0.14	0.18	−0.78	0.217
Non-zero	−0.45	0.68	−0.66	0.254
Autistic Student	−0.03	0.20	−0.15	0.440
Neurotype Match	0.17	0.12	1.48	0.069
Female	0.12	0.20	0.60	0.273
Mutuality	0.99	0.15	6.56***	< 0.001
Heterogeneity (Popularity)	−1.72	0.65	−2.63**	0.004
Heterogeneity (Activity)	−2.52	1.52	−1.65*	0.049
***Total*** (*N* = *7)*				
Sum (Intercept)	−0.99***	0.27	−3.62	< 0.001
Non-zero	0.02	0.77	0.02	0.490
Autistic Student	0.07	0.06	1.07	0.142
Neurotype Match	0.16***	0.05	3.52	< 0.001
Female	−0.02	0.12	−0.13	0.45
Mutuality	2.63***	0.25	10.40	< 0.001
Popularity	−1.21	1.34	−0.90	0.183
Activity	−1.29*	0.70	−1.83	0.033

Significance levels: *** < 0.001, ** < 0.01, * < 0.05. N = the number of ERGMs included in the meta-analysis. Average Estimate = weighted least squares estimator of average effect size (logit).

## Discussion

The purpose of this study was to examine the effect of student-peer neurotype match on peer relationships among autistic and non-autistic youth in an inclusive educational setting. Consistent with our hypotheses, the findings show that results showed that (1) student-neurotype match predicted more and stronger peer connections in both autistic and non-autistic youth; (2) youth showed a stronger tendency to connect with their same-neurotype peers than peers with similar social activity and popularity; and, (3) student-peer neurotype match significantly predicted increased total strength of peer connections in students’ social networks above and beyond the effects of student neurotype, mutuality, and level of sociality, while student neurotype had no significant effects in all networks. These preliminary findings supported the double empathy problem theory and suggested that the social challenges experienced by autistic youth may result from mutual barriers that emerged in cross-neurotype social interactions between autistic and non-autistic youth.

This study provided a new understanding of the social challenges among autistic youth in integrated learning environments, specifically on the role of peer neurotype. The findings showed that peers’ neurotype may have a stronger effect on autistic youth’s peer relationships than their autistic neurotype, suggesting that these youth’s peer challenges may not fully result from individual social deficits. These findings aligned with studies investigating the role of interpersonal contexts on social interactions among autistic and non-autistic adults, which revealed better social outcomes - than cross-neurotype (Crompton et al., 2020a,b; Morrison et al., 2020b). These studies, along with our findings, revealed positive outcomes in within-neurotype interactions among autistic individuals, contradicting the dominant narrative that autistic people’s social difficulties are mainly contributed by internal impairments. Supporting this, Morrison et al. (2020a) found that social cognition and social skills only minimally predict social outcomes among autistic adults. Collectively, these studies point to the need to consider alternative, interpersonal factors contributing to autistic people’s social challenges, such as the double empathy problem and non-autistic people’s perceptions and attitudes toward autistic people (Sheppard et al., 2016; Alkhaldi et al., 2019; Heasman and Gillespie, 2019; Morrison et al., 2019).

The study showed that student-peer neurotype match predicted the strength of peer relationships among both autistic and students above and beyond student neurotype and social network dynamics. Interestingly, student-peer neurotype match did not predict peer relationships in social initiation networks after controlling for other social network effects. This finding suggests that youth may not have *a priori* inclination of initiating interactions with a same-neurotype peer, rather, social challenges emerged in the interaction process following the initiations. According to the double empathy problem theory, both autistic and non-autistic youth may experience increased difficulties in negotiating mutual understanding and social interests in cross-neurotype interactions, which consequently shortens or terminated their interactions. The insignificant effect of neurotype match on social initiations in this study seems to depart from the findings of Morrison et al. (2020b), which suggest that neurotype match predicts better interests for future interaction among autistic and non-autistic adults. This discrepancy may be explained by differences between the social contexts of the studies. Different from participants in Morrison et al.’s study who engaged in dyadic interactions with an assigned partner, youth in our study interacted with peers in a school club setting, where social partners were partially determined by the availability of peers when in need of assistance. For example, a student might reach out to a cross-neurotype peer who is nearby to request assistance, while such interaction might not develop into a strong peer connection. Another potential explanation for this difference may be the age of participants, since adults may have developed strong social preference or biases toward atypical behavioral traits than youth.

The findings of this study differ from prior research investigating the social networks of autistic students in integrated education, which identified fewer peer connections and more peripheral social status among autistic students than non-autistic students (Chamberlain et al., 2007; Rotheram-Fuller et al., 2010; Kasari et al., 2011; Locke et al., 2012; Anderson et al., 2016). In addition to the fact that prior studies did not account for peer neurotype, this inconsistent finding may be due to differences in the methods to identify peer relationships and observation settings. While this study operationalized peer relationships as observed, frequent social interactions, prior studies mostly used a friend nomination method, where students were asked to identify their friends or the peers whom they would play with. This methodological difference likely leads to different findings, and we believe that both the subjective measure and objective observations provide a meaningful understanding of this research topic. Regarding the research setting, we investigated peer interaction networks in an integrated school club with fewer students and an equal autistic to non-autistic student ratio. Alternatively, previous studies examined friendship networks in bigger, mainstreamed classrooms, where autistic students were minorities. This difference in student compositions may greatly affect students’ social outcomes, as mainstreamed classrooms were dominated by non-autistic students and provided few chances for autistic within-neurotype connections. This potential reason for inconsistent findings indicates the need to investigate the role of peer neurotype compositions in studies of peer relationships among autistic students.

The findings of this study must be interpreted in the context of its limitations. As mentioned above, this study did not measure the youth’s subjective perceptions of friendships, and thus the observations may not represent their perceived peer relationships profiles. However, this study examined social interaction rates among the youth, which can serve as a helpful indicator for objective peer engagement and connections. In our observations, no negative or aggressive social behaviors were identified, and thus all social behaviors were included in the analysis for peer relationships. Secondly, this preliminary study used a small, homogeneous sample of students, and the findings may not generalize to the heterogeneous population and other settings. The small sample size for the social network modeling may result in underpowered estimations, even though there is currently no unambiguous notion of effective sample size for network analysis given the complexity of the data properties, distribution, and model assumptions (Krivitsky and Kolaczyk, 2015). Thirdly, we were not able to investigate the effect of matched gender on peer relationships in addition to matched neurotype with our sample because there was only one autistic girl in this study (i.e., it was not possible for the autistic girl to interact with another autistic girl). Lastly, the research was conducted in a school club, which does not represent the social interaction experience of autistic youth in all integrated education settings, such as in a classroom. We encourage future research to continue investigating the effects of peer factors on autistic social outcomes with a larger, more diverse sample across multiple settings. Additionally, we recommend future research to further investigate the mechanisms underlying the barriers in cross-neurotype peer interactions, as well as strategies to support these interactions. For example, a recent study by Silver and Parsons (2022) explored the social strategies that autistic adults identify to help their conversations with non-autistic social partners, providing useful knowledge to support mutual understanding in cross-neurotype interactions.

The study has implications for research and clinical practice. The findings on the effects of student-peer neurotype match on peer relationships suggest a need for autism research and interventions to shift the focus from individual social differences to the interpersonal barriers between autistic and non-autistic students. Instead of building normative social skills, alternative social support may include providing opportunities to build within-neurotype peer connections in extracurricular programs or peer support groups. The findings demonstrate the social potential of autistic students in an inclusive environment with a balanced composition of neurotypes, supporting an alternative model of social support in inclusive education. Future research and interventions may explore facilitators and barriers to mutual understanding between autistic and non-autistic individuals, such as factors associated with social perceptions, attitudes, and environments.

## Data availability statement

The datasets presented in this article are not readily available because the participants of this study were minorities. Requests to access the datasets should be directed to Y-LC, yulun.chen@nyu.edu.

## Ethics statement

The studies involving human participants were reviewed and approved by New York University and NYC Department of Education. Written informed consent to participate in this study was provided by the participants’ parents or legal guardians.

## Author contributions

Y-LC conducted data collection and analysis and completed the first draft of the manuscript. MS reviewed and contributed to the research design, data analysis, and manuscript development. KP acquired research funding and contributed to research and manuscript development. All authors contributed to the article and approved the submitted version.

## Conflict of interest

The authors declare that the research was conducted in the absence of any commercial or financial relationships that could be construed as a potential conflict of interest.

## Publisher’s note

All claims expressed in this article are solely those of the authors and do not necessarily represent those of their affiliated organizations, or those of the publisher, the editors and the reviewers. Any product that may be evaluated in this article, or claim that may be made by its manufacturer, is not guaranteed or endorsed by the publisher.

## Footnotes

1 We use the identity first language (i.e., autistic people) instead of the person first language (people with autism) because the former is preferred by a larger portion of the autistic community (Kenny et al., 2016).

2 Social activity and popularity were specified as within-actor uncentered covariances of square roots of out- and in-dyad values, respectively, which increase with great heterogeneity (Krivitsky, 2012).

## References

[B1] Alkhaldi R. S. Sheppard E. Mitchell P. (2019). Is there a link between autistic people being perceived unfavorably and having a mind that is difficult to read? *J. Autism Dev. Disord.* 49 3973–3982. 10.1007/s10803-019-04101-1 31197637 PMC6751138

[B2] Anderson A. Locke J. Kretzmann M. Kasari C. (2016). Social network analysis of children with autism spectrum disorder: predictors of fragmentation and connectivity in elementary school classrooms. *Autism* 20 700–709. 10.1177/1362361315603568 26567264 PMC4917452

[B3] Barrat A. Barthélemy M. Pastor-Satorras R. Vespignani A. (2004). The architecture of complex weighted networks. *Proc. Natl. Acad. Sci. U.S.A.* 101 3747–3752. 10.1073/pnas.0400087101 15007165 PMC374315

[B4] Bates D. Maechler M. Bolker B. Walker S. (2015). Fitting linear mixed-effects models using lme4. *J. Stat. Softw.* 67 1–48. 10.18637/jss.v067.i01

[B5] Bolis D. Balsters J. Wenderoth N. Becchio C. Schilbach L. (2017). Beyond autism: introducing the dialectical misattunement hypothesis and a Bayesian account of intersubjectivity. *Psychopathology* 50 355–372. 10.1159/000484353 29232684

[B6] Chamberlain B. Kasari C. Rotheram-Fuller E. (2007). Involvement or isolation? The social networks of children with autism in regular classrooms. *J. Autism Dev. Disord.* 37 230–242. 10.1007/s10803-006-0164-4 16855874

[B7] Chen Y. L. Senande L. L. Thorsen M. Patten K. (2021). Peer preferences and characteristics of same-group and cross-group social interactions among autistic and non-autistic adolescents. *Autism* 25 1885–1900. 10.1177/13623613211005918 34169757 PMC8419288

[B8] Cochran W. G. (1954). The combination of estimates from different experiments. *Biometrics* 10 101–129.

[B9] Cook A. Ogden J. Winstone N. (2016). The experiences of learning, friendship and bullying of boys with autism in mainstream and special settings: a qualitative study. *Br. J. Spec. Educ.* 43 250–271. 10.1111/1467-8578.12143

[B10] Cresswell L. Hinch R. Cage E. (2019). The experiences of peer relationships amongst autistic adolescents: a systematic review of the qualitative evidence. *Res. Autism Spectr. Disord.* 61 45–60. 10.1016/j.rasd.2019.01.003

[B11] Crompton C. J. Sharp M. Axbey H. Fletcher-Watson S. Flynn E. G. Ropar D. (2020a). Neurotype-matching, but not being autistic, influences self and observer ratings of interpersonal rapport. *Front. Psychol.* 11:586171. 10.3389/fpsyg.2020.586171 33192918 PMC7645034

[B12] Crompton C. J. Ropar D. Evans-Williams C. V. Flynn E. G. Fletcher-Watson S. (2020b). Autistic peer-to-peer information transfer is highly effective. *Autism* 24 1704–1712. 10.1177/1362361320919286 32431157 PMC7545656

[B13] Crompton C. J. Hallett S. Ropar D. Flynn E. Fletcher-Watson S. (2020c). ‘I never realised everybody felt as happy as i do when i am around autistic people’: a thematic analysis of autistic adults’ relationships with autistic and neurotypical friends and family. *Autism* 24 1438–1448. 10.1177/1362361320908976 32148068 PMC7376620

[B14] Csardi G. Nepusz T. (2006). The igraph software package for complex network research. *InterJ. Complex Syst.* 1695 1–9.

[B15] De Jaegher H. (2013). Embodiment and sense-making in autism. *Front. Integr. Neurosci.* 7:15. 10.3389/fnint.2013.00015 23532205 PMC3607806

[B16] Freeman L. C. (1978). Centrality in social networks conceptual clarification. *Soc. Netw.* 1 215–239. 10.1016/0378-8733(78)90021-7

[B17] Goodreau S. M. Kitts J. A. Morris M. (2009). Birds of a feather, or friend of a friend? Using exponential random graph models to investigate adolescent social networks. *Demography* 46 103–125. 19348111 10.1353/dem.0.0045 PMC2831261

[B18] Handcock M. Hunter D. Butts C. Goodreau S. Krivitsky P. Morris M. (2020). *ergm: Fit, Simulate and Diagnose Exponential-Family Models for Networks. R Package Version 3.11.0.* 10.18637/jss.v024.i03 PMC2743438 19756229

[B19] Heasman B. Gillespie A. (2019). Participants over-estimate how helpful they are in a two-player game scenario toward an artificial confederate that discloses a diagnosis of autism. *Front. Psychol.* 10:1349. 10.3389/fpsyg.2019.01349 31244739 PMC6579835

[B20] Hedges L. V. Olkin I. (2014). *Statistical Methods for Meta-Analysis.* Amsterdam: Elsevier Science.

[B21] Hedley D. Uljarević M. Wilmot M. Richdale A. Dissanayake C. (2018). Understanding depression and thoughts of self-harm in autism: a potential mechanism involving loneliness. *Res. Autism Spectr. Disord.* 46 1–7. 10.1016/j.rasd.2017.11.003

[B22] Holland P. W. Leinhardt S. (1981). An exponential family of probability distributions for directed graphs. *J. Am. Stat. Assoc.* 76 33–50.

[B23] Humphrey N. Symes W. (2011). Peer interaction patterns among adolescents with autistic spectrum disorders (ASDs) in mainstream school settings. *Autism* 15 397–419. 10.1177/1362361310387804 21454385

[B24] Hunter D. Handcock M. Butts C. Goodreau S. Morris M. (2008). ergm: a package to fit, simulate and diagnose exponential-family models for networks. *J. Stat. Softw.* 24 1–29. 10.18637/jss.v024.i03 19756229 PMC2743438

[B25] Jaswal V. K. Akhtar N. (2018). Being vs. appearing socially uninterested: challenging assumptions about social motivation in autism. *Behav. Brain Sci.* 42 1–73. 10.1017/S0140525X18001826 29914590

[B26] Kasari C. Locke J. Gulsrud A. Rotheram-Fuller E. (2011). Social networks and friendships at school: comparing children with and without ASD. *J. Autism Dev. Disord.* 41 533–544. 10.1007/s10803-010-1076-x 20676748 PMC3076578

[B27] Kenny L. Hattersley C. Molins B. Buckley C. Povey C. Pellicano E. (2016). Which terms should be used to describe autism? Perspectives from the UK autism community. *Autism* 20 442–462. 10.1177/1362361315588200 26134030

[B28] Krivitsky P. N. (2012). Exponential-family random graph models for valued networks. *Electron. J. Stat.* 6 1100–1128. 10.1214/12-EJS696 24678374 PMC3964598

[B29] Krivitsky P. N. Kolaczyk E. D. (2015). On the question of effective sample size in network modeling: an asymptotic inquiry. *Stat. Sci*. 30, 184–198. 10.1214/14-STS502 26424933 PMC4584154

[B30] Locke J. Kasari C. Rotheram-Fuller E. Kretzmann M. Jacobs J. (2012). Social network changes over the school year among elementary school-aged children with and without an autism spectrum disorder. *Sch. Ment. Health* 5 38–47. 10.1007/s12310-012-9092-y

[B31] Locke J. Shih W. Kretzmann M. Kasari C. (2016). Examining playground engagement between elementary school children with and without autism spectrum disorder. *Autism* 20 653–662. 10.1177/1362361315599468 26341991 PMC4779076

[B32] Lubbers M. J. (2003). Group composition and network structure in school classes: a multilevel application of the p* model. *Soc. Netw.* 25 309–332. 10.1016/s0378-8733(03)00013-3

[B33] Lusher D. Koskinen J. Robins G. (2013). *Exponential Random Graph Models for Social Networks : Theory, Methods, and Applications.* Cambridge: Cambridge University Press.

[B34] Maiano C. Normand C. L. Salvas M. C. Moullec G. Aime A. (2016). Prevalence of school bullying among youth with autism spectrum disorders: a systematic review and meta-analysis. *Autism Res.* 9 601–615. 10.1002/aur.1568 26451871

[B35] Mazurek M. O. (2014). Loneliness, friendship, and well-being in adults with autism spectrum disorders. *Autism* 18 223–232. 10.1177/1362361312474121 24092838

[B36] Milton D. E. M. (2012). On the ontological status of autism: the ‘double empathy problem’. *Disabil. Soc.* 27 883–887. 10.1080/09687599.2012.710008

[B37] Milton D. E. M. Heasman B. Sheppard E. (2020). “Double empathy,” in *Encyclopedia of Autism Spectrum Disorders*, ed. Volkmar F. (New York, NY: Springer), 1–9.

[B38] Morrison K. E. DeBrabander K. M. Faso D. J. Sasson N. J. (2019). Variability in first impressions of autistic adults made by neurotypical raters is driven more by characteristics of the rater than by characteristics of autistic adults. *Autism* 23 1817–1829. 10.1177/1362361318824104 30848682

[B39] Morrison K. E. DeBrabander K. M. Jones D. R. Faso D. J. Ackerman R. A. Sasson N. J. (2020b). Outcomes of real-world social interaction for autistic adults paired with autistic compared to typically developing partners. *Autism* 24 1067–1080. 10.1177/1362361319892701 31823656

[B40] Morrison K. E. DeBrabander K. M. Jones D. R. Ackerman R. A. Sasson N. J. (2020a). Social cognition, social skill, and social motivation minimally predict social interaction outcomes for autistic and non-autistic adults. *Front. Psychol.* 11:591100. 10.3389/fpsyg.2020.591100 33324295 PMC7723837

[B41] Newman M. E. J. (2002). Assortative mixing in networks. *Phys. Rev. Lett.* 89:208701. 10.1103/PhysRevLett.89.208701 12443515

[B42] Newman M. E. J. (2003). Mixing patterns in networks. *Phys. Rev. E* 67:026126. 10.1103/PhysRevE.67.026126 12636767

[B43] R Core Team (2020). *R: A Language and Environment for Statistical Computing.* Vienna: R Foundation for Statistical Computing.

[B44] Robins G. Pattison P. Kalish Y. Lusher D. (2007). An introduction to exponential random graph (p*) models for social networks. *Soc. Netw.* 29 173–191. 10.1016/j.socnet.2006.08.002

[B45] Rotheram-Fuller E. Kasari C. Chamberlain B. Locke J. (2010). Social involvement of children with autism spectrum disorders in elementary school classrooms. *J. Child Psychol. Psychiatry* 51 1227–1234. 10.1111/j.1469-7610.2010.02289.x 20673234 PMC2970745

[B46] Sheppard E. Pillai D. Wong G. T. Ropar D. Mitchell P. (2016). How easy is it to read the minds of people with autism spectrum disorder? *J. Autism Dev. Disord.* 46 1247–1254. 10.1007/s10803-015-2662-8 26603886

[B47] Silver K. Parsons S. (2022). Perspectives of autistic adults on the strategies that help or hinder successful conversations. *Autism Dev. Lang. Impair.* 7. 10.1177/23969415221101113 [Epub ahead of print]. PMC9620675 36382069

[B48] Snijders T. A. B. Baerveldt C. (2003). A multilevel network study of the effects of delinquent behavior on friendship evolution. *J. Math. Sociol.* 27 123–151. 10.1080/00222500305892

[B49] Wickham H. (2016). *ggplot2: Elegant Graphics for Data Analysis.* New York, NY: Springer-Verlag.

[B50] Williams E. I. Gleeson K. Jones B. E. (2019). How pupils on the autism spectrum make sense of themselves in the context of their experiences in a mainstream school setting: a qualitative metasynthesis. *Autism* 23 8–28. 10.1177/1362361317723836 29139322

